# Impact of Cadmium Stress on Growth and Physio-Biochemical Attributes of *Eruca sativa* Mill

**DOI:** 10.3390/plants11212981

**Published:** 2022-11-04

**Authors:** Abdul Waheed, Yakupjan Haxim, Waqar Islam, Mushtaq Ahmad, Sajjad Ali, Xuejing Wen, Khalid Ali Khan, Hamed A. Ghramh, Zhuqi Zhang, Daoyuan Zhang

**Affiliations:** 1 National Key Laboratory of Ecological Security and Resource Utilization in Arid Areas, Xinjiang Institute of Ecology and Geography, Chinese Academy of Sciences, Urumqi 830011, China; 2 Xinjiang Key Laboratory of Conservation and Utilization of Plant Gene Resources, Xinjiang Institute of Ecology & Geography, Chinese Academy of Sciences, Urumqi 830011, China; 3 Turpan Eremophytes Botanical Garden, Chinese Academy of Sciences, Turpan 838008, China; 4 Xinjiang Key Laboratory of Desert Plant Roots Ecology and Vegetation Restoration, Xinjiang Institute of Ecology and Geography, Chinese Academy of Sciences, Urumqi 830011, China; 5 Department of Zoology, Islamia College University, Peshawar 25120, Pakistan; 6 Department of Botany, Bacha Khan University, Charsadda 24461, Pakistan; 7 Unit of Bee Research and Honey Production, King Khalid University, Abha 61413, Saudi Arabia; 8 Applied College, Mahala Campus, King Khalid University, Abha 61413, Saudi Arabia; 9 Biology Department Faculty of Science, King Khalid University, Abha 61413, Saudi Arabia; 10 Binzhou Vocational College, Binzhou 256603, China

**Keywords:** heavy metals, Cd, photosynthesis, ROS, antioxidants

## Abstract

Plants may experience adverse effects from Cadmium (Cd). As a result of its toxicity and mobility within the soil-plant continuum, it is attracting the attention of soil scientists and plant nutritionists. In this study, we subjected young *Eruca sativa* Mill. seedlings to different levels of Cd applications (0, 1.5, 6 and 30 µmol/L) via pot experiment to explore its morpho-physio-biochemical adaptations. Our results revealed a significant Cd accumulation in leaves at high Cd stress. It was also demonstrated that Cd stress inhibited photosynthetic rate and pigment levels, ascorbate peroxidase (APX), guaiacol peroxidase (GPX), catalase (CAT), and superoxide dismutase (SOD) enzyme activities, and increased malondialdehyde (MDA) levels. Conversely, the concentration of total ascorbate (TAS) increased at all levels of Cd application, whereas that of ascorbic acid (ASA), and dehydroascorbate (DHA) increased at 1.5 (non-significant), 6, 30 and 6 µmol/L (significant), though their concentrations decreased non-significantly at 30 µmol/L application. In conclusion, Cd-subjected *E. sativa* seedlings diverted much energy from growth towards the synthesis of anti-oxidant metabolites and osmolytes. However, they did not seem to have protected the *E. sativa* seedlings from Cd-induced oxidative stress, causing a decrease in osmotic adjustment, and an increase in oxidative damage, which resulted in a reduction in photosynthesis and growth. Accordingly, we recommend that the cultivation of *E. sativa* should be avoided on soil with Cd contamination.

## 1. Introduction

Despite their importance as environmental pollutants, heavy metals pose a serious threat to soil and water quality, and plant and animal nutrition [1]. Heavy metals are a natural part of soils at trace levels. Mining, industry, and localized agriculture have contributed to toxic levels of metals in soils [2]. Several heavy metals have been accumulating in soils for a long time through industrial waste and sewage disposal, including Fe, Mn, Cu, Ni, Co, Cd, Zn, and Hg. The excess of some of these metals can adversely affect the growth, metabolism, physiology, and senescence of plants even though they are essential micronutrients supporting many regular processes in plants. [3,4]. Cd is of special concern due to its potential toxicity to biota at low concentrations. Low levels of Cd are particularly hazardous due to their possible toxic effects on flora and fauna. Despite its non-essential nature, Cd is harmful to all organisms [4].

Moreover, the concentrations of Cd also seem to interfere with their plant growth efficiency. Cd is mainly found in the earth’s crust and more properly in combination with Zn [5,6,7]. Cd is released into the environment by the Zn, Pb, and Cu industries, used in the application of phosphate-based fertilizers, urban composts, wastewater irrigation, and metalworking industries, and accumulates in many places. Among the toxic and non-essential environmental pollutants worldwide, Cd is a serious problem for agriculture due to its detrimental effects on crops. When plants grow in Cd-contaminated soil, their roots absorb the heavy metal, which builds up in different organs and eventually reduces plant growth [8,9,10,11].

Cd toxicity is known to induce oxidative stress, causing damage to the cellular membrane and electron transport and inhibiting/activating enzymes, and interfering with nucleic acids and photosynthesis, resulting in stunted growth [12,13]. Generally, oxidative stress is associated with the hyper-accumulation of reactive oxygen species such as superoxide ion (O_2_^•-^), hydrogen peroxide (H_2_O_2_), and hydroxyl radical (OHU^−•-^) that cause the conversion of fatty acids into toxic lipid peroxides and damage biological membranes [14,15,16]. On the other hand, nonradical reactive oxygen species (ROS) are often stable and have a longer half-life, including hypochlorous acid (HOCl), hydrogen peroxide (H_2_O_2_), singlet oxygen (^1^O_2_), and peroxynitrite (ONOO^−^). Almost all ROS are derived from O_2_^•-^. By reducing molecular oxygen by one electron, it is rapidly transformed into H_2_O_2_ by spontaneous or superoxide dismutase (SOD)-catalyzed dismutation [17,18]. Compared to other ROS, H_2_O_2_ has a long half-life, is water-soluble, and can easily diffuse within and between cells [19]. Secondary ROS (hypochlorous acid, hydroxyl radical, chloramines) accumulate and damage macromolecules. In excess ROS production, proteins, lipids, and DNA are oxidized, resulting in metabolic pathway alterations and cellular dysfunction, ultimately resulting in necrosis and apoptosis. Oxidative stress is widely recognized as the most important molecular mechanism responsible for Cd toxicity [20,21].

Under oxidative stresses, the physiology and biochemistry of plants are constantly changing to keep a balance between ROS and antioxidants. During oxidative stresses, ROS accumulation is an indication of stress persistence [21]. The ROS may compromise membrane integrity, cause elevated electrolyte leakage (E.L.), and lead to the oxidation of proteins and lipids [22,23]. However, to scavenge the higher levels of ROS, plants have an efficient defense system that includes enzymatic components like Glutathione reductase (GR), Glutathione Peroxidase (GPX), Catalase (CAT), and Ascorbate peroxidase (APX) and non-enzymatic components reduced glutathione (GSH); ascorbate, and proline) [24,25,26]. It has been extensively reported that oxidative stresses including the Cd toxicity induced higher proline levels in plants including the Brassicaceae family. It has been demonstrated that heavy metals adversely affect plant developmental activities in many cultivars of plants [18,19], however, the effect of Cd toxicity in *Eruca sativa* (taramira) was not studied.

The leafy vegetable taramira (*E. sativa*) is an important oilseed crop and a member of the *Brassicaceae* family. Originating in North Africa and the south of Europe, it is also grown in Canada, China, Germany, France, Poland, Sweden, and to some extent in India and Pakistan [18]. Several European countries consume it extensively, including Italy and Turkey [19,25]. Depending on its importance, it is essential to investigate its physiological responses under heavy metal-induced oxidative stress. Henceforth, this study was designed to evaluate the physio-biochemical mechanisms of *E. sativa* (taramira) under various concentrations of Cd. This study will be helpful to figure out the Cd-tolerability levels of *E. sativa* for practical implementation in natural field conditions.

## 2. Results

### 2.1. Impact of Cd Application on Growth of E. sativa

**Figure 1:**
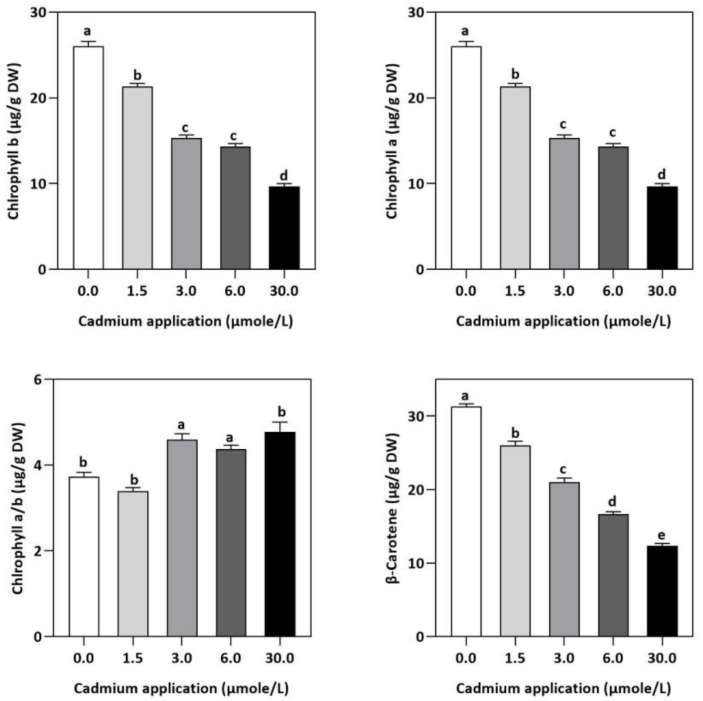
The photosynthesis pigment concentration in *E. sativa* leaves after Cd application. NSD, no significant detection. Each data value is presented at mean ± SE (n = 5). Under Tukey’s multiple range test, different letters indicate significant differences in the columns (*p* < 0.05).

**Figure 2:**
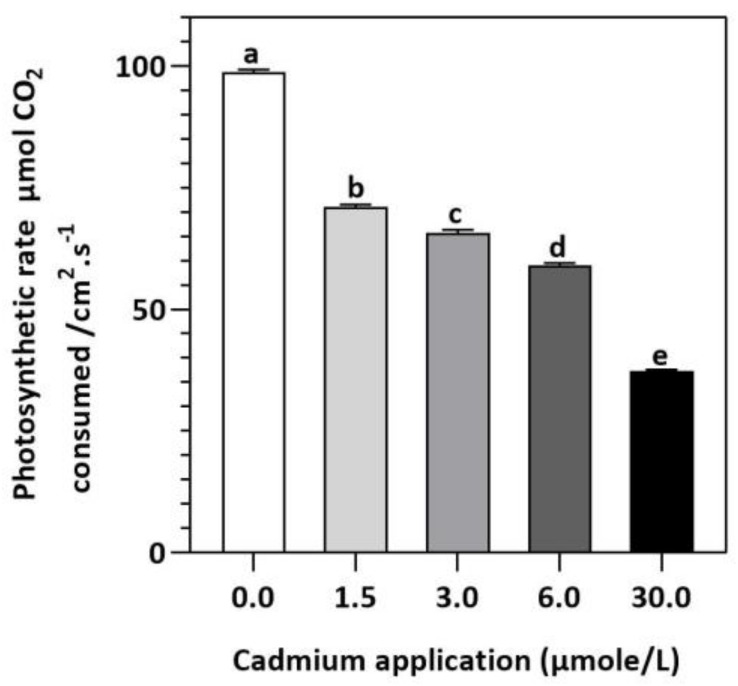
E. sativa photosynthesis rate after Cd application. Each data value is presented at mean ± SE (n = 5). Under Tukey’s multiple range test, different letters indicate significant differences (*p* < 0.05).

Cd-application significantly impacted the growth of *E. sativa* plants (Figure 1 and Figure 2). The shoot length and root length were significantly inhibited by all levels of Cd-application (Table 1). For instance, at 1.5, 3, 6, and 30 µmol/L of Cd-application significantly reduced the shoot length (19.38, 29.44, 39.50, and 44.47%, respectively) and root length (36.16, 45.57, 49.10, and 51.92%, respectively) with increasing Cd-application (Table 1).

### 2.2. Impact of Cd Application on Leaf Relative Water Content and Endogenous Cd Accumulation

It was found that the LRWC of *E. sativa* did not change significantly at 1.5 mol/L Cd application, but decreased by 19.69, 56.52, and 78.57% with the progression of Cd-doses at 3, 6, and 30 µmol/L, respectively (Table 2). Additionally, endogenous Cd accumulation was also enhanced with increasing Cd-application as compared to control (Table 1).

### 2.3. Influence of Cd Application on Photosynthetic Pigments and β-Carotene

In contrast to untreated plants, our results showed obvious differences between photosynthetic pigment levels in *E. sativa* plants treated with different levels of exogenous Cd (Figure 1). For instance, at 1.5, 3, 6, and 30 µmol/L of Cd application, the concentration of Chl-a decreased by 25.43, 27.49, 35.40, and 52.58%, respectively, whereas the Chl-b concentration decreased by 17.95, 41.03, 44.87, and 62.82%, respectively. Moreover, it was noticed that the impact of Cd-application was more pronounced on Chl-a than Chl-b (Figure 1). Therefore, the ratio of Chl-a to Chl-b considerably increased with increasing Cd-application, by 9.12, 22.95, 17.19 and 27.55% at 1.5, 3, 6, and 30 µmol/L of Cd, respectively (Figure 1). Moreover, based on the light-dependent CO_2_ consumption as a function of leaf area (Figure 2), the rate of photosynthesis was inhibited significantly with increasing Cd application levels in E. sativa seedlings as indicated by the 28.16, 33.56, 40.30, and 62.23% reduction after 1.5, 3, 6, and 30 µmol/L Cd application, respectively (Figure 2).

### 2.4. Impact of Cd Application on Malondialdehyde, Enzymatic Activities, and Metabolite Levels

**Figure 3:**
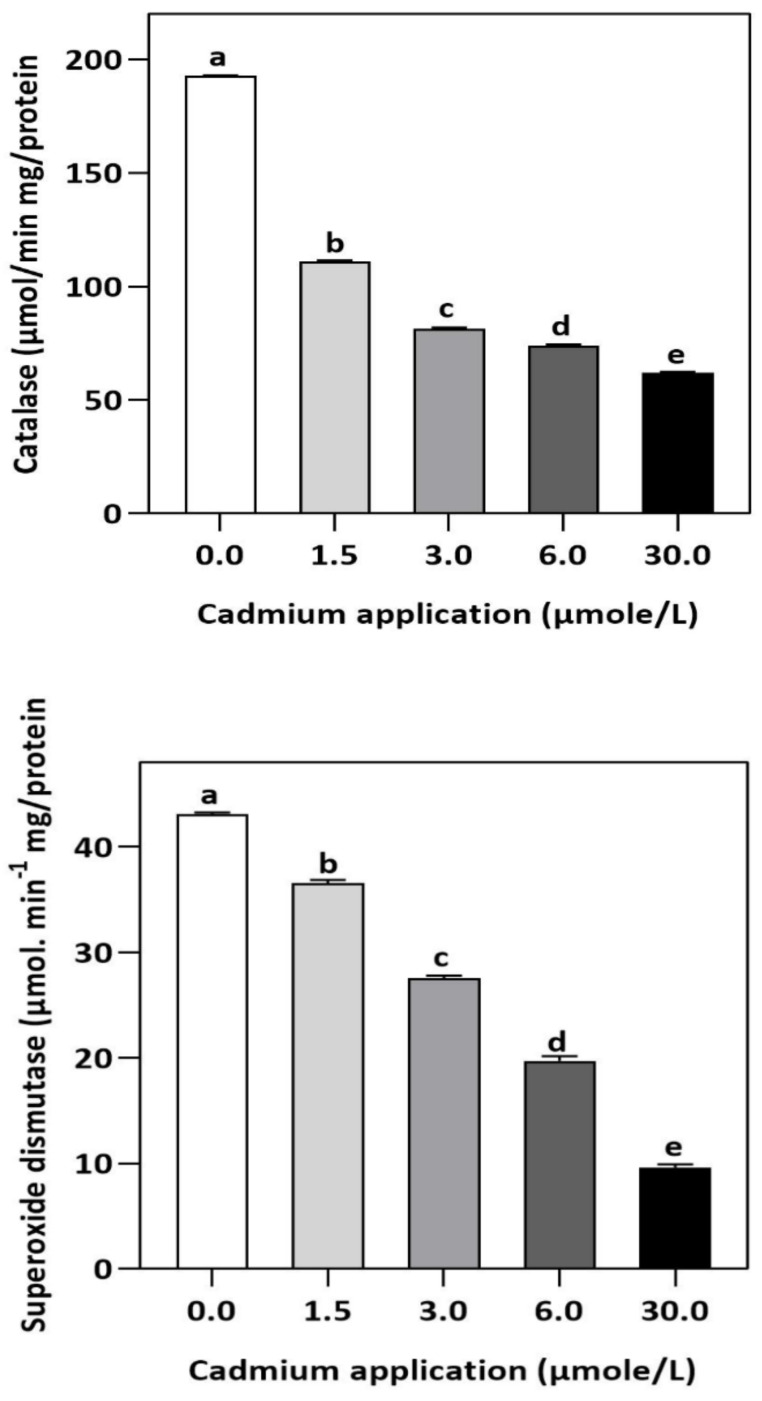
Effect of Cd application on the enzymatic activities of CAT and SOD in *E. sativa* leaves. Each data value is presented at mean ± SE (n = 5). In Tukey’s multiple range test (*p* < 0.05), different letters indicate significant differences in the columns.

**Figure 4:**
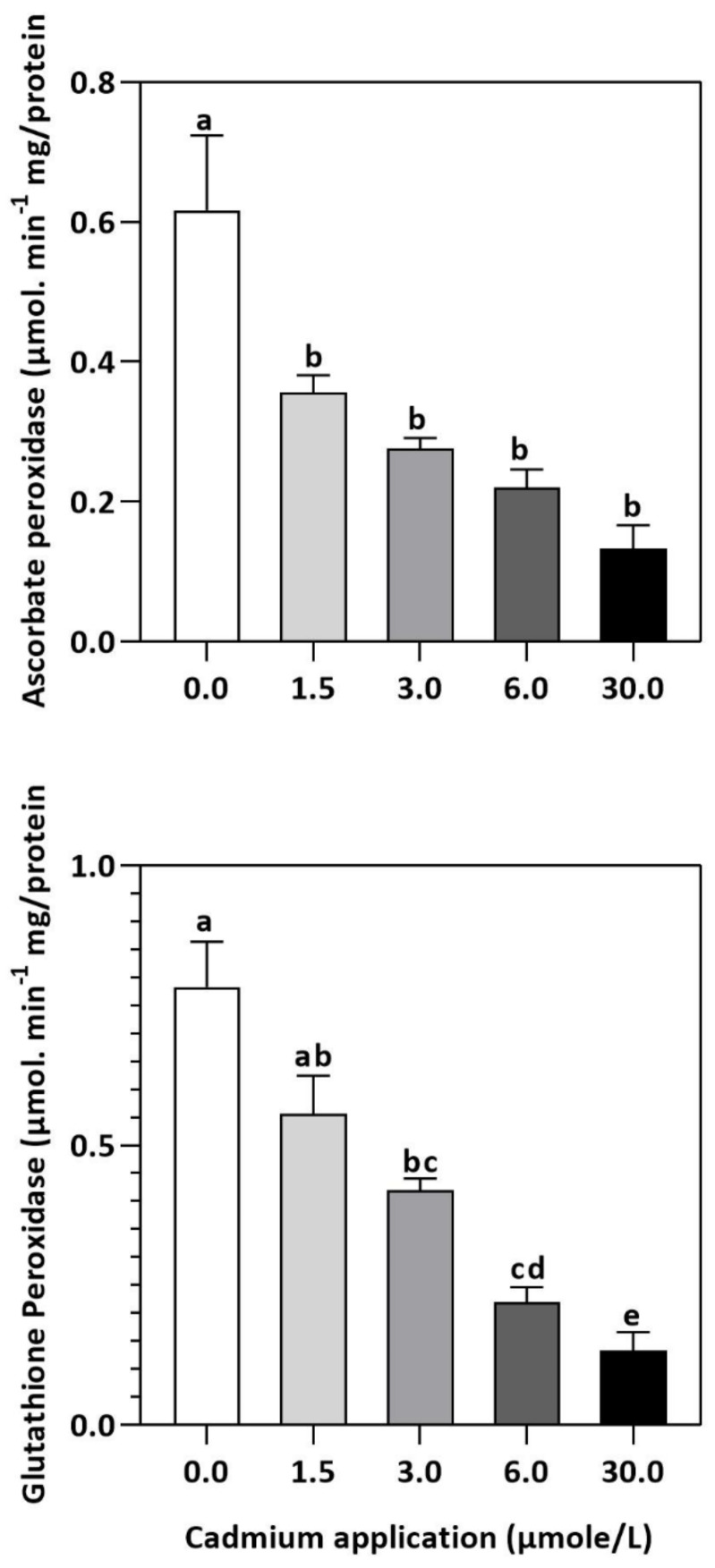
Inhibition of GPX and APX activities by Cd application in *E. sativa* leaves. Each data value is presented at mean ± SE (n = 5). In Tukey’s multiple range test (*p* < 0.05), different letters indicate significant differences in the columns.

**Figure 5:**
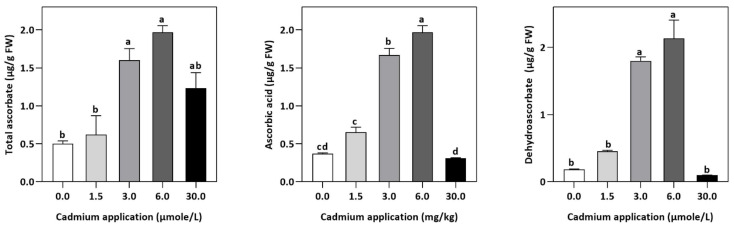
Effect of Cd application on the concentration of TAS, DHA, and ASA *E. sativa* leaves. Each data value is presented at mean ± SE (n = 5). In Tukey’s multiple range test (*p* < 0.05), different letters denote significant differences in columns.

**Figure 6:**
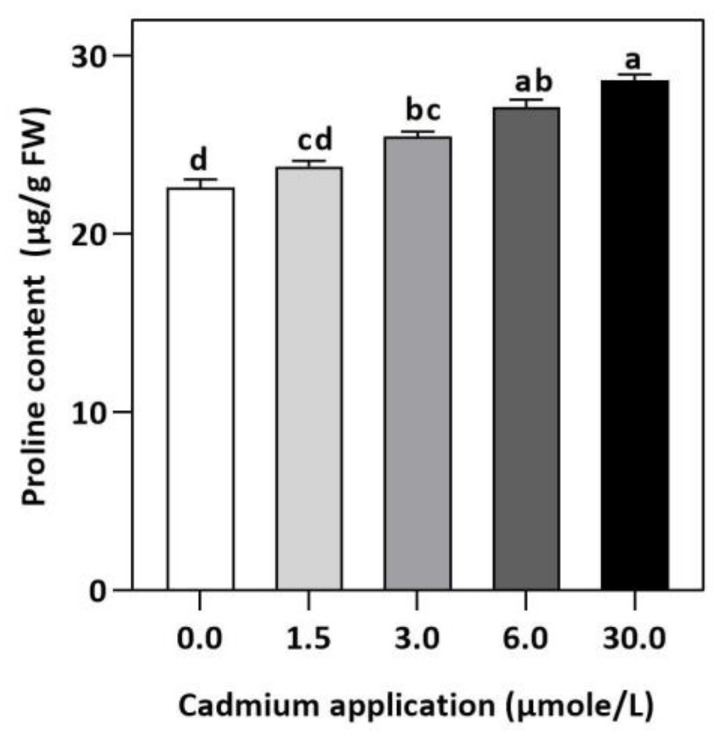
Effect of Cd application of on concentration of Proline *E. sativa* leaves. Each data value is presented at mean ± SE (n = 5). In Tukey’s multiple range test (*p* < 0.05), different letters denote significant differences in columns.

In plants, an increase in malondialdehyde (MDA) is a sign of oxidative damage. The cd-induced proliferation of MDA was observed in leaves of *E. sativa*. The concentration of MDA is up-regulated with the increasing Cd-application. The maximum concentration of MDA was exhibited by the highest application of Cd (30 µmol/L) (Table 2). In response to oxidative damage, plants produce various antioxidant enzymes and metabolites to protect themselves from damaging effects. Increasing levels of Cd-application increased non-protein thiol concentrations in our study. Moreover, a significant decrease in SOD and CAT enzyme activities was observed after Cd application (Table 2). After 1.5, 3, 6, and 30 µmol/L Cd application, the CAT activity was decreased by 42.39, 57.84, 61.67, and 67.75%, respectively, whereas the reduction of 15.16, 36.22, 54.12, and 77.65% was experienced in SOD activity, respectively. Collectively, the Cd application had a greater impact on CAT than SOD (Figure 3). Further, the activity of the APX and GPX enzymes decreased significantly with increasing Cd-application. Interestingly, the levels of the antioxidant metabolites were up-regulated with increasing Cd-application (Figure 4). Regardless of the amount of Cd applied, the total ascorbate (TAS) concentration was enhanced. Conversely, the concentrations of ASA, (Figure 5). Dehydroascorbate (DHA), and proline levels were up-regulated until 6 µmol/L Cd, while its decline was seen after 30 µmol/L of Cd-application (Figure 5 and Figure 6).

### 2.5. Relationship between the Studied Parameters

**Figure 7:**
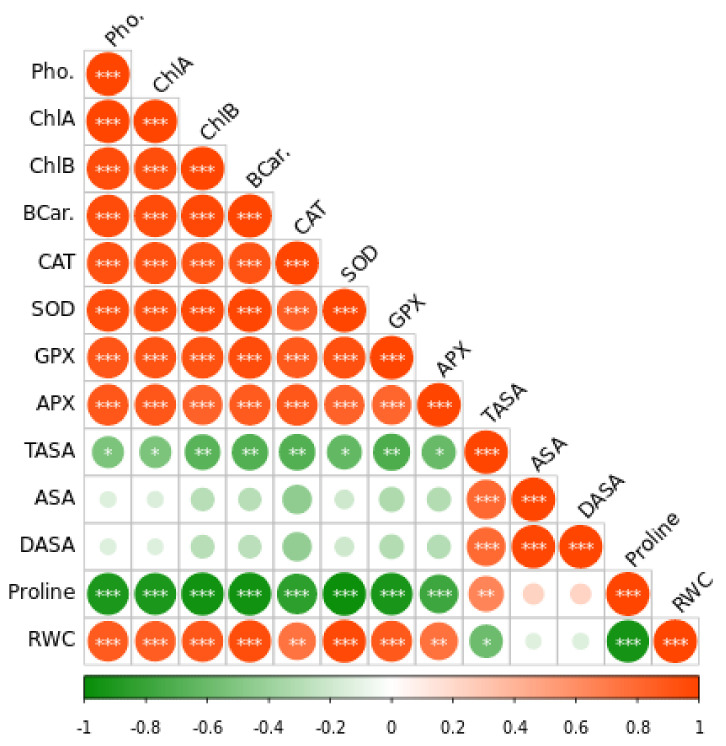
The Pearson correlation matrix for leaf relative water content (RWC), dehydroascorbate (DHA), reduced ascorbate (ASA), total ascorbate (TASA), ascorbate peroxidase (APX), glutathione peroxidase activity (GPX), superoxide dismutase (SOD), catalase (CAT) photosynthesis (Pho). Pearson’s correlation was calculated and heat maps were visualized by R Statistical computing software (version 4.1.3). (*) *p* < 0.05; (**) *p* < 0.01; (***) *p* < 0.001.

According to Pearson correlation analysis, oxidative stress-associated parameters negatively correlated with antioxidants and photosynthesis rate (Figure 7) inferring that plants produce more antioxidants by increasing photosynthesis to reduce the Cd-induced oxidative stress.

## 3. Discussion

During their life, plants have to cope with various environmental constraints including heavy metals stress in particular. For instance, plants growing in soil with high Cd-level experience alterations in metabolic functions leading to stunted growth and productivity worldwide. In our study, Cd accumulation in leaves of *E. sativa* increased with increasing Cd-application as compared to control, which could be attributed to impaired metabolism and growth of young *E. sativa* seedlings. For example, accumulation of Cd within plant organs had been reported to negatively interfere with essential physiological processes and plant growth [27,28,29]. The abrupt negative impacts of Cd accumulation are correlated highly to its mobility in plant tissues [30]. We found that Cd stress negatively affected the growth and metabolism of *E. sativa*. A decrease in shoot length and root length was observed with increasing levels of Cd stress. Plant photosynthetic organs and structures were damaged by Cd stress, which could explain the reduction in *E. sativa* growth [31].

In addition, Cd stress affects plant mineral uptake by limiting the acquisition of essential minerals [32], leading to reduced plant growth. Moreover, Cd stress can decline or inhibit the process of photosynthesis, which reduces the production of photo-assimilates which ultimately decreases their growth; these negative effects are generally more pronounced on plant aboveground organs than underground organs [33]. As a result, leaf relative water content (LRWC) is a reliable indicator of a plant’s ability to handle stress. In the present study, the LRWC in *E. sativa* seedlings under Cd-stress was significant decreased in comparison with the controlled seedlings [34].

Moreover, Cd-stress had obvious effects on the concentration of photosynthetic pigments in *E. sativa* leaves in comparison with their control. For instance, the concentration of chlorophyll suddenly decreased with increasing Cd levels. A plethora of studies had reported the reduction in chlorophyll concentration of other crop plants such as tomato [35,36], barley [37], maize [38], garden cress [27], and mustard [39] subjected to Cd stress. A negative impact of Cd-stress on iron (Fe) concentration has been reported in leaves, causing chlorophyll metabolism to be impaired [40]. Furthermore, chlorophyll degradation and/or inhibition of its biosynthesis were reported to contribute to impaired photosynthesis in Cd-stressed plants [41]. Additionally, we also noticed increasing Cd-stress had more significant effects on Chl-b than Chl-a, leading to an increased ratio of Chl-a to -b, indicating the susceptibility of chlorophyll-b to Cd-stress. Therefore, we speculated that increasing Cd stress negatively impacted the photosynthetic capacity of *E. sativa*, leading to reduced growth.

A negative effect of Cd toxicity on plants’ physiological mechanisms has also been reported [42]. Overproduction of ROS can, for example, lead to lipid peroxidation, membrane damage, and enzyme inactivation, which adversely affects the cell’s performance and viability by interacting with plant metabolites such as proteins, lipids, nucleic acids, and other important substances [43]. Abiotic stress causes plants to produce ROS, or reactive oxygen species, which activate their organs and protect them from damaging effects. One of the most abundant and stable ROS is H_2_O_2_, which plays a key regulatory role in the healthy functioning of their organs [44,45]. As a result of various external stimuli, MDA levels in cellular organelles are generally increased. MDA is an indicator of lipid peroxidation within cellular organelles [46].

In our study, increasing Cd stress led to an increased concentration of MDA in the leaves of *E. sativa* seedlings. It is speculated that Cd-stress induced significant peroxidation of the inner membrane of *E. sativa* seeds, resulting in damage to their membrane structure [47]. Conversely, plants generally increase their anti-oxidant enzyme activities, thereby decreasing the peroxidation of lipid membranes and thus maintaining biological membrane integrity [48]. The primary enzymes involved in this protective mechanism include SOD, POD, and CAT. However, in our study, Cd-stress decreased the anti-oxidant enzymes, indicating the high susceptibility of *E. sativa* to Cd-stress-induced oxidative damage. Interestingly, we noticed a gradual decrease in anti-oxidant enzymes with increasing Cd stress, indicating a threshold beyond which the *E. sativa* seedlings were not able to modulate the allocation of energy and resources for maintaining the enzymatic anti-oxidant mechanism. Phytochemical properties, as well as the concentration or properties of the heavy metal, affect antioxidant enzymes’ resistance to heavy metal stress [47]. Therefore, we speculate that Cd-stress caused the reduction in the activities of the antioxidant enzyme activities and consequent protection from oxidative stress damage [49,50].

Meanwhile, APX plays a crucial role in removing H_2_O_2_, but its activity is dependent on metal concentrations, and its primary purpose is to eliminate H_2_O_2_ at the source of generation [51,52]. The formation of GPX can be induced by heavy metal toxicity and is more effective than CAT at eliminating H_2_O_2_ [53,54]. Therefore, CAT, GPX, and APX, enzymes are also considered to play key functions in the elimination of cellular H_2_O_2_ concentrations in plants [55]. The decrease in CAT activity in leaves of *E. sativa* is also accompanied by reduced APX and GPX activity, indicating further susceptibility of young *E. sativa* seedlings to Cd-induced oxidative stress damage [56].

Despite the anti-oxidant enzymes, plants also produce an array of non-enzymatic anti-oxidant metabolites for scavenging the over-production of ROS. In our study, non-enzymatic antioxidants, such as TAS, ASA, and DHA, significantly increased with increasing Cd stress, which is in line with previous findings [57,58]. Ascorbate protects the plant from oxidative stress. For instance, it protects metabolic processes against H_2_O_2_ and ROS [59]. Moreover, non-thiol showed upward trends with increasing Cd stress. It has been demonstrated that non-protein thiols can help plants detoxify heavy metals by containing a high amount of cysteine sulfhydryl residues. Our results are consistent with previous findings of Cd-subjected, *Arabidopsis thaliana,* and *Brassica oleracea* var. *acephala* [60,61].

In addition to maintaining cell function, protecting the membrane structure of cells, and maintaining the stability of biological macromolecules, proline plays an essential osmotic protective role [62]. As a result of heavy metal stress, plants’ water balance is negatively affected and Proline levels are increased, which is crucial for cell osmotic regulation [63]. Plants produce a large amount of Proline in response to abiotic stresses [64,65] and accumulate Proline in their organs as a result of a wide range of abiotic stresses. There are different pathways for synthesizing and degrading Proline in plants under different conditions, so Proline’s effects are not completely consistent [66,67]. *E. sativa* synthesized more proline under Cd-stress in order to resist osmotic stress, as evidenced by the increasing proline concentration when Cd levels increased. Our results exhibited that although a higher accumulation of non-enzymatic anti-oxidant metabolites somehow took into account the function of eliminating ROS, however, they were not able to fully exercise the anti-oxidant mechanism because of reduced antioxidant enzymes, leading to higher lipid peroxidation and impaired photosynthesis and growth. Therefore, our study highly discourages the cultivation of *E. sativa* Cd-contaminated soil.

## 4. Conclusions

In our study, the increasing Cd-stress levels significantly decreased the plant growth as indicated by reduced shoot and root growth. However, the significantly increased Cd-accumulation in leaves with increasing Cd-stress indicated that *E. sativa* is a good candidate for the phytoremediation of Cd-polluted soil. Moreover, the photosynthesis rate and chlorophyll pigments concentration decreased with increasing Cd-stress. In addition, Cd stress enhanced lipid peroxidation whereas the antioxidant mechanism showed a differential response. Increasing Cd-stress resulted in the reduction of SOD and GPX, CAT, and APX activities. Conversely, the concentration of total ascorbate, ascorbic acid, and dehydroascorbate increased under Cd application. Moreover, the thiols and proline levels were also increased with increasing Cd stress. However, Cd-application diminished the physio-biochemical mechanism of *E. sativa* seedlings, resulting in a loss of osmotic adjustment, an increase in oxidative damage, and a reduction in photosynthesis and growth. Hence, the cultivation of *E. sativa* should be avoided on soils contaminated with Cd. However, further molecular and proteomics studies are required to get deeper insights into the Cd-stress tolerance by *E. sativa*.

## 5. Material and Methods

### 5.1. Plant Material and Growing Conditions

We obtained taramira (*E. vesiceria* subsp. *sativa*) seeds from the National Agricultural Research Center (NARC), Islamabad, Pakistan. Plants were grown in plastic pots filled with soil (the soil pH was 6.02, and the total amount of Cd in the soil was 0.87 mg kg^−1^) having two small holes at the bottom. We rotated all pots regularly in a greenhouse environment with temperatures between 18 and 25 °C and humidity levels between 60 and 70% during the day and night, respectively. A respective concentration of Cd was added to each treatment group. We divided the pots into five treatments, i.e., T1 (Controlled, distilled water with no Cd), T2 (1.5 µmol/L), T3 (3 µmol/L), T4 (6 µmol/L), and T5 (30 µmol/L). Each treatment was replicated five times. The roots and shoots of control and Cd-exposed plants were collected after 20 days of exposure to different Cd applications. A measurement of the root and shoot length was performed. In order to determine the dry weight (D.W.) of the leaves, we first measured the fresh weight of the leaves and then dried them at 70 °C for three days. Fifteen different plants were studied.

### 5.2. An Analysis of the Relative Water Content (RWC)

The relative water content (RWC) was determined using the standard method described in [68] and calculated using the following Equation.

RWC= [(f.m.-d.m.)/(f.s.m-d.m.)] × 100.

After floating samples in distilled water at 20 °C for 4 h in darkness, the fully saturated mass (f.s.m.) was determined.

### 5.3. Determination of Foliar Cadmium Content

Plant samples were tested for Cd concentrations. The taramira leaf samples were washed with deionized water and dried at 105 °C for 60 min followed by 60 °C for 24 h. They were then ground and sieved through a nylon sieve of 1 mm size. The reaction was carried out with a ratio of 3:1, *v*/*v*, of HNO_3_:HClO_4_ in the microwave using a gram of samples. An atomic absorption spectrophotometer (3300) from Perkin-Elmer was used to measure Cd concentrations. For quality assurance, Cd was included as a standard material. Cd concentrations were averaged based on triplicate analyses. This analysis indicated a detection limit of 0.05 µg/dw for Cd [69,70].

### 5.4. Photosynthetic Rate and Chlorophyll Content Determination

The chlorophyll a, chlorophyll b, and β-carotenes contents of leaves were examined by the previously described methods [71,72]. Furthermore, we measured the rate of photosynthesis, based on the light-dependent CO_2_ consumption/m^2^ leaf area from excised leaves at 30 °C, a standard method (YSI 5300A, Yellow Springs Instruments, OH, USA) was followed using a Biological Oxygen Monitor (YSI 5300A).

### 5.5. Analyzing Antioxidant Enzyme Activity

In order to homogenize the leaves (0.2 g), glass powder and a prechilled mortar and pestle were used together with 50 mM K-phosphate buffer (pH 7.0), which contained 1 mM ethylene diamine tetraacetic acid (EDTA) and 1% (*w*/*v*) insoluble polyvinyl polypyrrolidone (PVPP). A 20 min centrifugation at +4 °C at 15,000× *g* was performed after homogenization. To measure enzyme activity, supernatants were stored at –20 °C [73].

As a measure of CAT activity, H_2_O_2_ was degraded over two minutes at 240 nm in the absence of any supernatant. Each enzyme’s specific activity was expressed by the amount of H_2_O_2_ oxidized per mg of protein per minute. We measured the activity of SOD as described by [74] and expressed it in enzyme units as described by Madhava Rao and Srestry [75].

In order to measure the activity of GPX, the oxidation of guaiacol was measured (extinction coefficient 25.5 mM^−1^ cm^−1^) following a standard method described by Kato and Shimizu [54]. Furthermore, the enzymatic activity was expressed as moles of H_2_O_2_ reduced per mg−1 of protein.

With minor modifications, the APX was measured according to the method of Nakano and Asada [76]. Observing an extinction coefficient of 2.8 mM^−1^ cm^−1^ at 290 nm (excitation 280 nm) for five minutes revealed an extinction coefficient of 2.8 mM^−1^ cm^−1^. The amount of ascorbate oxidized per minute per mg of protein was determined as mol ascorbate oxidized per minute per mg of protein.

### 5.6. Determination of Antioxidant Metabolites

The concentration of proline was determined following a standard method [77]. A spectrophotometer was used to measure the absorbance of the reaction mixture at 546 nm. Using a calibration curve for proline (Sigma), the concentration of proline was expressed as μmole proline (g F.W.^−1^)]. A standard method was used to measure the concentrations of soluble non-protein thiols and total ascorbate [78,79]. Using ascorbic acid as a reduction agent in an acidic solution, Fe^+3^ is reduced to Fe^+2^, while Ellman’s reagent was used to measure non-protein thiols [80]. To carry out the centrifugation, a Kubota Corporation, TOKYO model 5500 centrifuge was used. With a spectrophotometer model T70 (P.G. Instruments, Leicestershire, UK.), we conducted spectrophotometric measurements.

### 5.7. Statistical Analysis

Three measurements were repeated and data were organized using Microsoft Excel 2016 as mean values ± S.E. A one-way analysis of variance was performed using SPSS v16.0 (SPSS Inc., Chicago, IL, USA, 2007). To test for significance, we used Tukey’s multiple range test (*p* < 0.05), making pairwise comparisons of the mean values. Prior to fitting the ANOVA, the data were checked for normality and homogeneity of variance. The figures were drawn using GraphPad Prism 8.0, which displays bars as the mean ± S.E.

## Figures and Tables

**Table 1:** Changes in root and shoot lengths of *E. sativa* with respect to Cd applications.

Cd-Application (µmol/L)	Shoot Length (cm)	Root Length (cm)	Cd Uptake (μg/g DW)	Leaf Relative Water Content (%)
Control	26.83 ± 0.15 ^a^	21.57 ± 0.35 ^a^	0.00 ± 0.00 ^a^	90.23 ± 0.41 ^a^
1.5	21.63 ± 0.09 ^b^	13.77 ± 0.27 ^b^	0.08 ± 0.00 ^b^	89.13 ± 0.32 ^b^
3	18.93 ± 0.15 ^c^	11.74 ± 0.12 ^c^	0.10 ± 0.00 ^c^	72.47 ± 0.64 ^c^
6	16.23 ± 0.13 ^d^	10.98 ± 0.24 ^c,d^	0.18 ± 0.01 ^c^	39.23 ± 0.35 ^d^
30	14.90 ± 0.12 ^d^	10.37 ± 0.09 ^e^	0.26 ± 0.01 ^d^	19.33 ± 0.69 ^e^

NSD, no significant detection. Each data value is presented at mean ± SE (n = 5). Different letters above the columns denote significant differences under Tukey’s multiple range test (*p* <0.05) for significance.

**Table 2:** The concentration of MDA and non-protein thiol in *E. sativa* in response to Cd application.

Cd-Application (µmol/L)	MDA (µg·g^−1^ FW)	Non-Protein Thiol (µg·g^−1^ FW)
Control	25.15 ± 0.15 ^e^	0.02 ± 0.00 ^c^
1.5	38.52 ± 0.09 ^d^	0.02 ± 0.00 ^c^
3	29.58 ± 0.25 ^c^	0.03 ± 0.00 ^c^
6	43.70 ± 0.40 ^b^	0.04 ± 0.00 ^b^
30	49.23 ± 0.15 ^a^	0.06 ± 0.00 ^a^

Each data value is presented at mean ± SE (n = 5). In Tukey’s multiple range test (*p* < 0.05), different letters indicate significant differences in the columns.

## Data Availability

Not applicable.
